# Alexithymia and negative emotions among nursing students: a moderated mediation model

**DOI:** 10.1186/s12912-024-01832-0

**Published:** 2024-03-08

**Authors:** Feifei Sun, Fang Wang, Xiaojing Hu, Jiaomei Xue, Shangkun Zheng, Jing Su, Qinghua Lu

**Affiliations:** 1 Department of Nursing, Shandong Mental Health Center, Shandong University, 49 Wenhua East Road, 250014 Jinan, Shandong China; 2 Xianning Vocational Technical College, 437100 Xianning, Hubei China; 3 Society and Law School, Shandong Women’s University, Changqing University Science and Technology Park, No. 2399, University Road, 250300 Jinan, Shandong China; 4 Human Resources Department, Shandong Mental Health Center, Shandong University, 49 Wenhua East Road, 250014 Jinan, Shandong China; 5 Editorial Board, Journal of Shandong First Medical University, No. 6699 Qingdao Road, Huaiyin District, 250000 Jinan, China; 6 Department of Infection Management, Shandong Mental Health Center, Shandong University, 49 Wenhua East Road, 250014 Jinan, Shandong China

**Keywords:** Alexithymia, Negative emotions, Resilience, Perceived social support, Nursing students

## Abstract

Nursing students, who comprise a high percentage of China’s college students, experience many psychological problems; however, few studies explored the mechanisms underlying these problems. This cross-sectional study explored the relationships and mechanisms of depression, anxiety, stress, and narrative disorders in senior nursing students. Questionnaires were administered to 380 senior nursing students in Hubei Province using the Sociodemographic Questionnaire, Toronto Alexithymia-20 Scale, Perceived Social Support Scale, 10-Item Connor-Davidson Resilience Scale, and Depression-Anxiety-Stress Scale. After controlling for sociodemographic variables, Hayes’ PROCESS macros were used to test how psychological resilience moderates the relationships among narrative disorders, negative affect, and perceived social support. Bootstrap confidence intervals tested for indirect effects. Correlation analyses revealed that alexithymia was correlated significantly positively with depression-anxiety-stress (*r* = 0.57, 0.56, and 0.58, resp.) and significantly negatively with perceived social support (*r* = 0-0.46). Psychological resilience was correlated significantly negatively with alexithymia (*r*=-0.39) and depression-anxiety-stress (*r*=-0.31, -0.30, and-0.32, resp.) but significantly positively with perceived social support(*r* = 0.50). Perceived social support was correlated significantly negatively with depression-anxiety-stress (*r*=-0.33, -0.34, and − 0.42 resp.). Stress was correlated significantly positively with anxiety and depression (*r* = 0.81 and 0.77, resp.). Psychological resilience was a partial mediator between depression and dysphoria (β=-0.08, *p* < 0.05). Dysphoria directly predicted anxiety (β = 0.31) and stress (β = 0.37); moreover,alexithymia predicted depression not only directly but also through the mediating effect of psychological resilience. Therefore, educators and clinical administrators must promote and recognise negative emotions among nursing students to help ensure the nursing workforce’s stability.

## Background

Globally, more than 350 million people ($\sim$4.7% of the global population) have depression. Depression is the leading cause of disability worldwide and a major contributor to the global burden of disease, and anxiety disorders’ prevalence is estimated to be 7.3% worldwide [1]. Many individuals have comorbidities with these disorders, and their symptoms may be subsyndromal, residual, undiagnosed [2], or untreated [3]. With socioeconomic development, nursing personnel demand is increasing in China. Nursing students in senior vocational schools (hereafter ‘nursing students’) are an important reserve force for nursing positions in China, as they obtain special vocational education through coursework, skills training, and intense internship competition. They also experience high-level depression and anxiety [4]. Understanding nursing students’ internalisation problems and the factors affecting them is important to ensure their psychological health, successful study completion, and competent future nursing career. However, few studies have examined the psychosocial mechanisms of depression and anxiety in nursing students, although the results could provide targeted guidance for nursing students’ psychological and social improvement and a reduction in clinical risk of psychological disorders. This study addressed this research gap by examining the mediating and moderating relationships between narrative disorders; depression anxiety, and stress; and psychological resilience.

Previous studies have showed that narrative disorders are closely associated with psychopathology and emotional–behavioural problems [5]. Alexithymia is a personality trait characterised by difficulty in identifying and describing feelings, lack of ability to distinguish physiological arousal from feelings, and an externally oriented thinking style,It is often associated with negative mood states such as depression [6] and anxiety [7]. Research has showed that people with narrative disorders often misinterpret others’ emotions and fail to respond appropriately, causing social difficulties [8]. Moreover, alexithymia adversely affects patients’ stress-management methods [9]. Those who can sensitise, express, and adjust their emotions effectively are more successful in handling stress and emotional experiences [10]. Therefore, people with narrative disorders are at risk of difficulties in coping with stress, suggesting that affective disorders are potential risk factors and predictors of certain psychosomatic or psychiatric disorders, especially among nursing students [11]. Studies have showed that high-level narrative impairment is strongly associated with internalising problems, which predict individuals’ depression and anxiety levels [12]. People with narrative disorders are easily exhausted because of inadequate coping strategies, weak social support networks, and the inability to manage their own or others’ emotions [13]. Such indirect effects indicate that narrative disorders make people depressed more easily [11]. Furthermore, several studies have found that alexithymia, a specific disturbance in psychic functioning characterised by difficulties in verbalising affect and elaborate fantasies [14], is negatively correlated with psychological well-being [15]. People with high (vs. low) affective traits experience more interpersonal difficulties and mental health problems [16]. Therefore, lack of self-awareness of internal emotions (i.e., narrative disorders) is now considered a transdiagnostic mechanism to moderate mental health outcomes [17]. Several studies have noted that difficulty in recognizing emotions is related to the severity of anxiety symptoms and that narrative disorders are a predictor of health-related anxiety [18]. According to a recent study, narrative disorders are positively associated with depression, anxiety, and stress [19]. The research thus considers narrative affective disorders as a unifying construct, although its aspects and specific outcomes may have subtle differences (e.g., difficulties in describing and identifying emotions) [18]. Affective disorders may be precursors to the development of socio-emotional and mental health problems through difficulties in perceiving/producing emotions [20] and internal feelings (perception of internal body states and/or emotional regulation) [21]. However, the role of narrative disorders (and their individual aspects) in predicting the development of mental health problems remain unclear. These findings also echo the stress-representation disorder hypothesis that narrative disorders contribute to the development of stress-related disorders [22]. Affective disorders are primarily influenced by neurophysiological, social, and cognitive mechanisms. Regarding neurophysiological mechanisms, research shows that the prefrontal cortex and cingulate gyrus activities are similar in individuals with social anxiety, suggesting a relationship between affective disorders and social anxiety [23].

Moreover, the nursing curriculum itself may develop students’ emotional and empathic abilities to foster beneficial therapeutic relationships with patients and contact with patients’ distress may help develop and refine students’ emotional and empathic skills. Nursing students without appropriate training in managing the emotional care burden develop emotional withdrawal (a narrative disorder) as an implicit coping strategy. Therefore, the nursing curriculum should involve detecting and controlling narrative impairment because it can reduce students’ emotional competence and ability to establish appropriate therapeutic relationships with patients [24].

According to the resilience framework, psychological resilience is a complex dynamic phenomenon related to a person’s ability to overcome adversity, and it can play a mediating role in propelling a person to grow in the face of adversity [25].Resilience may be effective in reducing alexithymia and stress, and resilience mediated the relationship between dysphoria and stress among Chinese medical students during the New Crown Pneumonia pandemic [26]. Resilience in nursing students is a process with periods of engagement and disengagement and shows students that higher resilience is associated with fewer psychological barriers [27], lower psychological distress, and greater positive thinking among college and nursing students [28]. Moreover, nursing students’ resilience is significantly associated with positivity, persistence, and empathy [28], and is a consistently positive attribute that helps them adapt to typical learning environments and environment stressors. Therefore, focusing on nursing students’ resilience has positive implications for their physical and psychological health [29]. This study highlights alexithymia as a possible predictor of resilience. Further, narrative disorders correspond to an impaired understanding of emotions [30]. Some studies have reported that narrative impairment in patients with psychological disorders reduces patient resilience [31], and it is negatively associated with resilience among Chinese military personnel [32].

People with low psychological resilience may also experience depression, anxiety, and stress [5]. Studies on clinical and non-clinical populations found that psychological resilience, depression, and anxiety are related [33], and negative environmental influences can have serious deleterious effects on mental health outcomes. However, many individuals do not develop psychopathology despite a history of adversity, and research has increasingly focused on identifying factors associated with resilience that may counteract the deleterious effects of stress and compensate for biological risk conditions [34]. Consequently, a broad range of resilience-promoting characteristics and mechanisms were identified. For example, managing anxiety and stress using integrative approaches can build resilience in adolescents throughout their lives [35]. Nevertheless, there is a need to study nursing students’ situations, narrative disorders, and ability to cope with depression, anxiety, and stress. Accordingly, this study proposes that psychological resilience may mediate the relationship between narrative disorders and depression, anxiety, and stress. Within this theoretical framework, nursing students with narrative disorders may be prone to low psychological resilience [26], leading to depression, stress, and anxiety.

Perceived social support is an individuals’ perception of the amount and quality of support they receive from their social network [36]. Social support has been another focus in research on protective factors for depressive symptoms [37]. One study highlighted perceived social support as a possible protective factor for depression among occupational therapists in Hong Kong during the pandemic; higher levels of perceived social support were associated with lower levels of depression [38]. Stress, protective factors (e.g., resilience, social support), and psychological well-being among nursing students have been increasingly reported in the literature. However, there is scant research on the interactions between these variables in the later stages of the COVID-19 pandemic and in the context of developing countries. Nursing students face some level of psychological stress during both their schooling and clinical placements, which means that inadequate social support can increase their negative emotions [39]. A previous study showed that social support moderates the relationship between anxiety and psychological distress [40]. A Philippine study showed that social support mediates the effects of stress on resilience, positive thinking, and work capacity [41]. However, research is lacking on the perceived social support as a direct or indirect buffer to the effects of narrative disorders on negative emotions such as depression, anxiety, and stress in nursing student populations.

Alexithymia is negatively associated with and predicts perceived social support [42], meaning low perceived social support is significantly associated with high alexithymia. Individuals with alexithymia do not benefit from social support due to deficits in their emotional perception [43]. Perceived social support predicts personal well-being and is strongly associated with positive emotions, such as optimism, and personality traits, such as self-esteem [44]. Social support significantly predicts depression: the lower the perceived social support, the more severe the patients’ depressive symptoms [45]. Moreover, loneliness (i.e., anxiety and depression) is strongly associated with objective social isolation and perceived social support [46, 47]. Theoretical models suggest that loneliness has social, cognitive, and biological consequences that increase the risk of subsequent depression. Potential mechanisms for this association include negative perceptions of social interactions, negative cognitive patterns (e.g., low self-confidence), anticipation of social threats, increased stress, lower self-esteem, and biological effects on stress responses and inflammation [48]. Besides its effect on long-term mental health outcomes, loneliness may cause poor physical health outcomes and increased mortality in patients with severe mental health problems [49]. Moreover, social support may be an important factor influencing undergraduate students’ mental health [50] and psychological well-being. Yet, scant research has examined this among higher-level nursing students. Low social support and high-level alexithymia are associated with increased psychological distress, primarily through difficulties identifying feelings [51]. While alexithymia is associated with a lack of social support, its underlying mechanisms are not well understood.

**Fig. 1:**
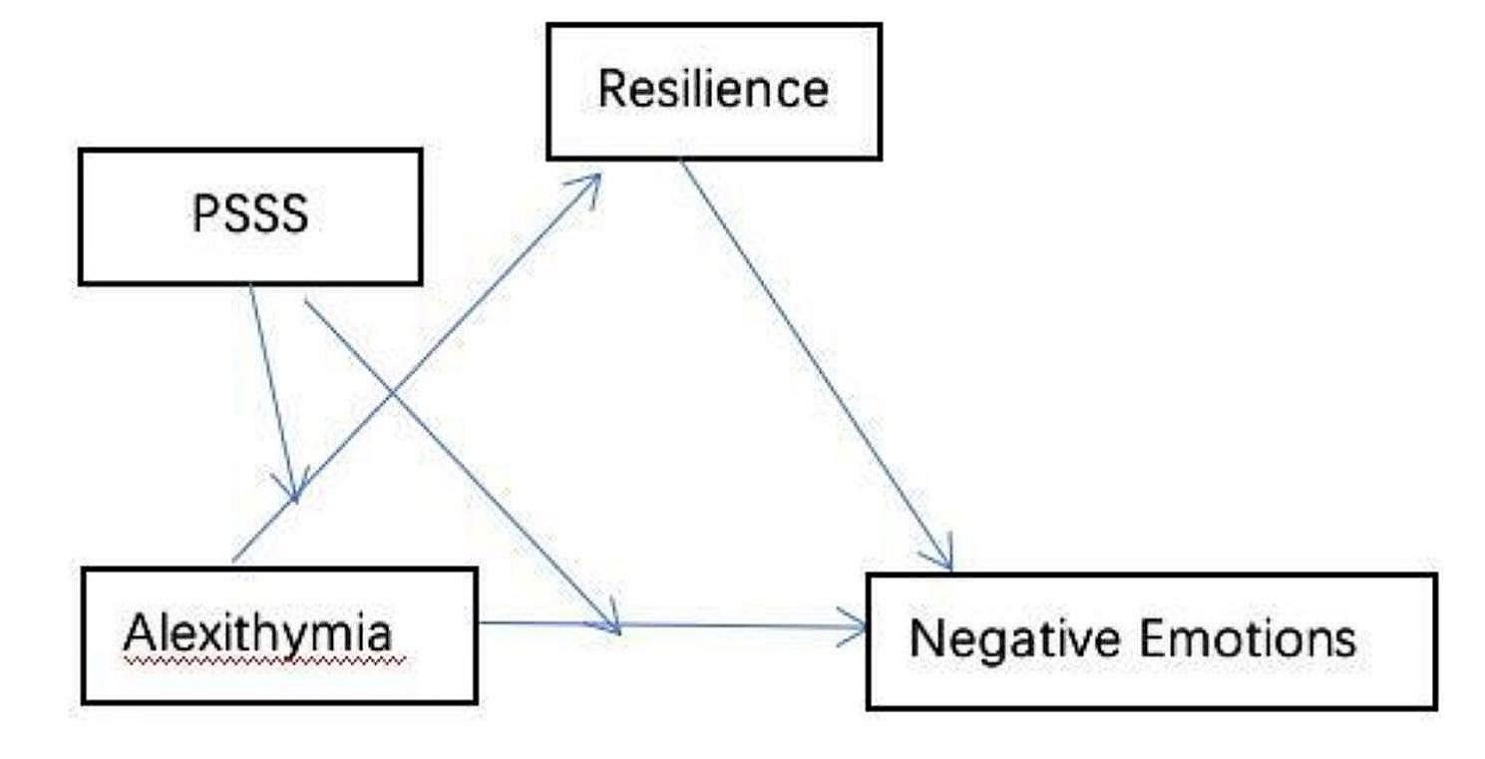
Moderated mediation models

Therefore, this study aimed to gain a more comprehensive understanding of the mechanisms underlying affective disorders and depression, anxiety, and stress in higher-education nursing students. Thus, it examines whether (1) narrative disorders are related to depressive anxiety; (2) psychological resilience mediates the association between narrative disorders and depressive anxiety; and (3) perceived social support moderates the pathway from narrative disorders to depressive anxiety through psychological resilience. The potential mediating and moderating mechanisms of narrative disorders and depression, anxiety, and stress formed a moderated mediation model (Fig. 1). Accordingly, three hypotheses are proposed:

### Hypothesis 1

Alexithymia, depression, anxiety, and stress are positively correlated among senior nursing students.

### Hypothesis 2

Psychological resilience plays a mediating role in the relationship between alexithymia and depression, anxiety, and stress.

### Hypothesis 3

Social support moderates the direct and indirect relationships between alexithymia and depression, anxiety, and stress through psychological resilience.

## Methods

### Study design

This study employed a cross-sectional survey design.

### Setting and participants

From 1 to 31 October, 2022, 380 nursing students were selected to participate in an anonymous survey using convenience sampling at a college of higher education in Hubei Province. They were full-time first-year nursing students with no history of mental illness and who spoke Mandarin. Those currently involved in other mental health-related research or taking antipsychotic medications.

were excluded. Minimum sample size was calculated using Power Analysis and Sample Size Software (PASS, version 11.0) with a two-sided 95% confidence interval (CI) of 1.5, mean-to-limit distance of 11.3, and an SD of 11.3 [52]. The minimum sample size required was 266 with a 20% shedding rate.We recruited 380 nursing students, all of whom met the study criteria and completed the self-administered questionnaire (response rate: 100.0%) in 20–30 min.

### Ethical considerations

All participants were informed about the study’s purpose and their rights before providing informed consent. All information remained confidential and anonymous, and participants were informed that they could withdraw participation at any time. This study was approved by the ethics committee and complied with the Declaration of Helsinki.

### Measures

Our study was conducted in the Nursing Department’s classroom through an electronic questionnaire on ‘Questionnaire Star’ and included the following sub-questionnaires, all of which were translated into Chinese and validated.

#### Sociodemographic questionnaire

Data were obtained using a self-designed questionnaire covering age, gender, and educational level; all items were self-assessed.

#### Toronto alexithymia-20 scale (TAS-20)

TAS-20 was developed by Taylor [14] with three dimensions and 20 questions, of which questions 4, 5, 10, 18, and 19 were reverse scored. Dimensions 1–3 represent the inability to discriminate emotions, inability to express emotions, and extroverted thinking, respectively. A five-point scale was used, ranging from 1 = strongly disagree to 5 = strongly agree. Higher scores indicate higher-level affective disorder. A total TAS score of ≥ 61 indicated more severe narrative disorders. The Chinese version’s reliability and validity [53] were good in both the general and clinical populations [54], comparable to the scale’s English version [55]. Cronbach’s α = 0.83 in this study.

#### Perceived social support scale (PSSS)

The PSSS, developed by Zimet et al. [56], is a 12-item self-report scale measuring respondents’ perceived support from family, friends, and significant others. It contains dimensions 1–3 for support from family, friends, and others, respectively. The PSSS is scored on a seven-point scale, ranging from 1 = strongly disagree to 7 = strongly agree; higher scores indicate higher-level social support. In this study, Cronbach’s α = 0.89. The PSSS’s Chinese version had adequate internal consistency among adolescents (Cronbach’s α = 0.84), with values of 0.81, 0.92, and 0.83, respectively for dimensions 1–3 [57]. In this study, the total scale’s Cronbach’s α = 0.96, and that for dimensions 1–3 was 0.96, 0.92, 0.92, and 0.92, respectively.

#### 10-Item connor-davidson resilience scale (CD-RISC-10)

The CD-RISC-10 is a simplified version of the Psychological Resilience Scale. The original CD-RISC-10 was developed by Campbell-Sills and Stein [58] in English. Wang et al. [59] translated it into Chinese to measure resilience. The CD-RISC-10 assesses individuals’ psychological resilience in various situations and uses a five-point scale, ranging from 0 = never to 4 = always. Higher scores indicate higher-level psychological resilience. The scale’s Cronbach’s α = 0.96 in this study.

#### Depression anxiety stress scale (DASS)

The DASS [60] has three subscales to assess individuals’ depression, anxiety, and stress. It has good construct and content validity [61] and includes 21 items: seven per subscale, scored on a four-point scale, with zero representing non-compliance and three representing best compliance. The sum of each subscale’s seven-item scores multiplied by two is the score of that subscale. The scores ranged from 0 to 42; higher scores indicate more severe depression, anxiety, or stress [62]. Cronbach’s α for the total scale was 0.94, and that for anxiety, depression, and stress was 0.83, 0.85, and 0.85, respectively.

### Data analysis

SPSS 26.0 (IBM Corp., Armonk, NY, USA) was used for descriptive statistical and correlation analyses. The mediation and moderated mediation model were analysed using the PROCESS macro for SPSS [63]. The bias-corrected 95% CI was calculated with 5,000 bootstrapping re-samples. With a confidence interval that does not include zero, a significant moderated mediation effect could be established [64].

## Results

We distributed 380 questionnaires and obtained 380 valid responses (response rate: 100%). The participants included 59 (15.5%) men and 321 (84.5%) women, aged 18–23 years (mean age = 20.01 ± 0.827; Table 1).

**Table 1:** Sociodemographic characteristics of the participants

Variables		N (380)	Prevalence (%)
Gender	Men	59	15.53
	Women	321	84.47
Education Level	College and below	374	98.42
	Bachelor’s degree	4	1.05
	Master’s degree	1	0.26
	Doctoral degree	1	0.26
Family’s economic situation	Below average	267	70.26
	Medium	111	29.21
	Upper medium	2	0.53
Only child	Yes	73	19.21
	No	307	80.79

### Correlation between alexithymia, psychological resilience, perceived social support, and negative emotions

The correlation analysis (Table 2) shows two significant correlations between narrative disorder, psychological resilience, and depression, anxiety, and stress. Narrative disorder was correlated significantly positively with depression, anxiety, and stress (*r* = 0.57, 0.56, 0.58, respectively) and significantly negatively with perceived social support (*r* = -0.46). Psychological resilience was correlated significantly negatively with narrative disorder (*r* = -0.39) and depression, anxiety, and stress (*r* = -0.31, -0.30, and − 0.32, respectively), and significantly positively with perceived social support (*r* = 0.50). Perceived social support was correlated significantly negatively with depression, anxiety, and stress (*r* = -0.33, -0.34, and − 0.42, respectively). Stress was correlated significantly positively with anxiety and depression (*r* = 0.81 and 0.77, respectively).

**Table 2:** Descriptive statistics and correlations for the main study variables (*N* = 380)

Variable	Mean	SD	1	2	3	4	5	6
1. Resilience	34.18	7.07	1.00					
2. Alexithymia	49.06	9.17	-0.39**	1.00				
3. PSSS	60.24	12.42	0.50**	-0.46**	1.00			
4. Stress	18.94	5.39	-0.31**	0.57**	-0.33**	1.00		
5. Anxiety	18.21	4.76	-0.30**	0.56**	-0.34**	0.81**	1.00	
6. Depression	17.95	5.12	-0.32**	0.58**	-0.42**	0.77**	0.81**	1.00

*Note*. **p < 0.01. SD = standard deviation; PSSS = Perceived Social Support Scale

#### Alexithymia and negative emotions: moderated mediation modelling

First, psychological resilience’s mediating effect on the relationship between dysphoria and depression was tested using Model 4 (a simple mediation model) in the SPSS macro developed by Hayes (2013) [63], controlling for gender and age. The results (Table 3) indicate that alexithymia is a significant predictor of depression (B = 0.0.32, t = 13.76, *p* < 0.01), and upon including the mediator variable, dysphoria’s direct predictive effect on depression remained significant (B = 0.30, t = 11.83, *p* < 0.01). Moreover, dysphoria’s negative predictive effect on psychological resilience was significant (B = -0.30, t = -8.04, *p* < 0.01) as was the negative predictive effect of psychological resilience on depression (B = -0.08, t = -2.53, *p* < 0.01). The upper and lower bounds of the bootstrap 95% CIs for dysphoria’s direct effect on depression and psychological resilience’s mediating effect did not contain zero (Table 4), suggesting that dysphoria not only directly predicted depression but also mediated psychological resilience’s effect in predicting depression. Psychological resilience’s mediating effect accounted for 7.57% of the total effect.

**Table 3:** PROCESS macro testing the mediating effect of psychological resilience between alexithymia and depression, test of the mediating effect of psychological resilience

Regression equation		Fit indicator					Significance	
Outcome variable	Predictor variable	Coeff.	SE	R	R^2^	F	t	p
Depression	Gender	-0.13	0.60				-0.22	0.83
	Age	-0.12	0.26				-0.47	0.64
	Alexithymia	0.32	0.02	0.58	0.34	63.47	13.76	0.00
Resilience	Gender	-0.77	0.93				-0.83	0.40
	Age	-0.29	0.41				-0.72	0.47
	Alexithymia	-0.30	0.04	0.39	0.15	22.59	-8.04	0.00
Depression	Gender	-0.20	0.59				-0.33	0.74
	Age	-0.15	0.26				-0.56	0.57
	Alexithymia	0.30	0.03	0.59	0.35	49.89	11.83	0.00
	Resilience	-0.08	0.03				-2.53	0.01
Anxiety	Gender	-0.27	0.91				-0.30	0.76
	Age	0.33	0.17				1.92	0.06
	Alexithymia	0.31	0.03	0.55	0.31	46.38	11.49	0.00
Resilience	Gender	1.44	1.06				-0.83	0.41
	Age	0.28	0.20				-0.72	0.47
	Alexithymia	-0.28	0.03	0.46	0.21	27.87	-8.96	0.00
Anxiety	Gender	-0.15	0.91				-0.33	0.74
	Age	0.35	0.17				-0.56	0.57
	Alexithymia	0.29	0.03	0.56	0.31	35.79	0.26	0.79
	Resilience	-0.08	0.05				9.49	0.00
Stress	Gender	0.02	1.05				0.01	0.99
	Age	0.42	0.20				2.13	0.03
	Alexithymia	0.37	0.03	0.57	0.33	50.80	12.01	0.00
Resilience	Alexithymia	1.44	1.06				1.36	0.18
	Gender	0.28	0.20				1.39	0.16
	Age	-0.28	0.03	0.46	0.21	27.87	-8.96	0.00
Stress	Alexithymia	0.12	1.05				0.12	0.91
	Gender	0.44	0.20				2.23	0.03
	Age	0.35	0.03	0.58	0.33	38.68	10.11	0.00
	Alexithymia	-0.08	0.06				-1.37	0.17

*Note*. SE = standard error

**Table 4:** Mediating role of psychological resilience in the relationship between alexithymia and depression, alexithymia and anxiety and alexithymia and stress

	Total	B	SE	t	p	LLCI	ULCI
Alexithymia and Depression	Total	0.32	0.02	13.76	0.00	0.28	0.37
	Direct	0.30	0.03	11.83	0.00	0.25	0.35
	Indirect	0.02	0.01			0.01	0.05
Alexithymia and Anxiety	Total	0.31	0.03	11.49	0.00	0.26	0.36
	Direct	0.29	0.03	9.49	0.00	0.23	0.35
	Indirect	0.02	0.02			-0.01	0.06
Alexithymia and Stress	Total	0.37	0.03	12.01	0.00	0.31	0.43
	Direct	0.35	0.03	10.11	0.00	0.28	0.42
	Indirect	0.02	0.02			-0.02	0.06

Note: SE = standard error; LLCI = lower limit confidence interval; ULCI = upper limit confidence interval

Psychological resilience’s mediating effect on the dysphoria–anxiety relationship, controlling for gender and age, was examined using Model 4 in the SPSS macro developed by Hayes [63]. The results show that alexithymia was a significant predictor of anxiety (B = 0.31, t = 11.49, *p* < 0.01), and psychological resilience had a significant negative predictive effect on anxiety (B = -0.08, t = 9.49, *p* < 0.01). When the mediator variable was included, dysphoria’s negative predictive effect on anxiety was not significant (B = 0.28, t = 0.26, *p* = 0.79). The upper and lower bounds of the bootstrap 95% CIs for psychological resilience’s mediating effect contained zero (Table 4), suggesting that dysphoria directly predicted anxiety, and psychological resilience did not directly mediate the dysphoria–anxiety relationship.

Psychological resilience’s mediating effect on the dysphoria–stress relationship, controlling for gender and age, was examined using Model 4 in the SPSS macro. Table 3 shows that alexithymia was a significant predictor of stress (B = 0.37, t = 12.01, *p* < 0.01), and psychological resilience was a significant negative predictor of stress (B = -0.28, t = -8.96, *p* < 0.01). When mediator variables were included, dysphoria’s negative predictive effect on stress was not significant (B = -0.08, t = -1.37, *p* = 0.17). Upper and lower bounds of the bootstrap 95% CI for psychological resilience’s mediating effect contained a zero (Table 4), indicating that dysphoria directly predicted stress, and psychological resilience did not mediate the dysphoria–anxiety relationship.

Second, the moderated mediation model was tested by controlling for gender and age using Model 8 in the SPSS macro developed by Hayes (2013; Model 8 assumes that the mediation model’s first half and direct path are moderated, consistent with this study’s theoretical model).

Table 5 shows that product terms of narrative disorders and perceived social support are significant predictors of both psychological resilience and depression when perceived social support is included (depression: B = -0.01, t = -6.65, *p* < 0.01; psychological resilience: B = -0.01, t = -5.00, *p* < 0.05), suggesting that perceived social support can predict depression in the contexts of both narrative disorders and depression. Perceived social support moderates alexithymia’s direct prediction of depression.

**Table 5:** PROCESS macro testing the perceived social support in the moderated mediation model, depression

Moderated mediation analysis (depression)		Fitting indicator							Significance	
Outcome variable	Predictor variables	Coeff.	SE	LLCI	ULCI	R	R^2^	F	t	p
Resilience	Constant	40.59	7.64	25.57	55.60	0.57	0.33	36.18	5.32	0.00
	Alexithymia	-0.14	0.04	-0.22	-0.07				-3.87	0.00
	PSSS	0.22	0.03	0.17	0.28				8.18	0.00
	Alexithymia×PSSS	-0.01	0.00	-0.02	-0.01				-5.00	0.00
PSSS	Constant	22.21	5.27	11.85	32.58	0.66	0.43	47.51	4.21	0.00
	Alexithymia	0.27	0.03	0.22	0.32				10.65	0.00
	Resilience	-0.10	0.03	-0.16	-0.03				-2.76	0.01
	PSSS	-0.06	0.02	-0.10	-0.02				-3.15	0.00
	Alexithymia×PSSS	-0.01	0.00	-0.02	-0.01				-6.65	0.00
Moderated mediation analysis (anxiety)	constant	27.98	4.10	19.92	36.04	0.62	0.39	39.33	6.83	0.00
Resilience	Alexithymia	-0.15	0.03	-0.22	-0.09				-4.76	0.00
	PSSS	0.22	0.03	0.17	0.28				8.46	0.00
	Alexithymia×PSSS	-0.01	0.00	-0.01	-0.01				-5.68	0
PSSS	Constant	15.17	4.15	6.99	23.34	0.58	0.34	26.65	3.65	0.00
	Alexithymia	0.27	0.03	0.21	0.34				8.71	0.00
	Resilience	-0.13	0.05	-0.23	-0.02				-2.38	0.02
	PSSS	0.01	0.03	-0.05	0.06				0.31	0.75
	Alexithymia×PSSS	-0.01	0.00	-0.01	0.00				-3.42	0.00
Moderated mediation analysis (stress)	Constant	27.98	4.10	19.92	36.04	0.62	0.39	39.33	6.83	0.00
Resilience	Alexithymia	-0.15	0.03	-0.22	-0.09				-4.76	0.00
	PSSS	0.22	0.03	0.17	0.28				8.46	0.00
	Alexithymia×PSSS	-0.01	0.00	-0.01	-0.01				-5.68	0.00
PSSS	Constant	14.16	4.85	4.62	23.69	0.59	0.35	27.36	2.92	0.00
	Alexithymia	0.34	0.04	0.27	0.42				9.36	0.00
	PSSS	-0.12	0.06	-0.24	0.00				-1.89	0.06
	Alexithymia×PSSS	0.01	0.03	-0.05	0.08				0.41	0.68
	Constant	-0.01	0.00	-0.01	0.00				-2.62	0.01

*Note*. SE = standard error; LLCI = lower limit confidence interval; ULCI = upper limit confidence interval; PSSS = Perceived Social Support Scale

**Fig. 2:**
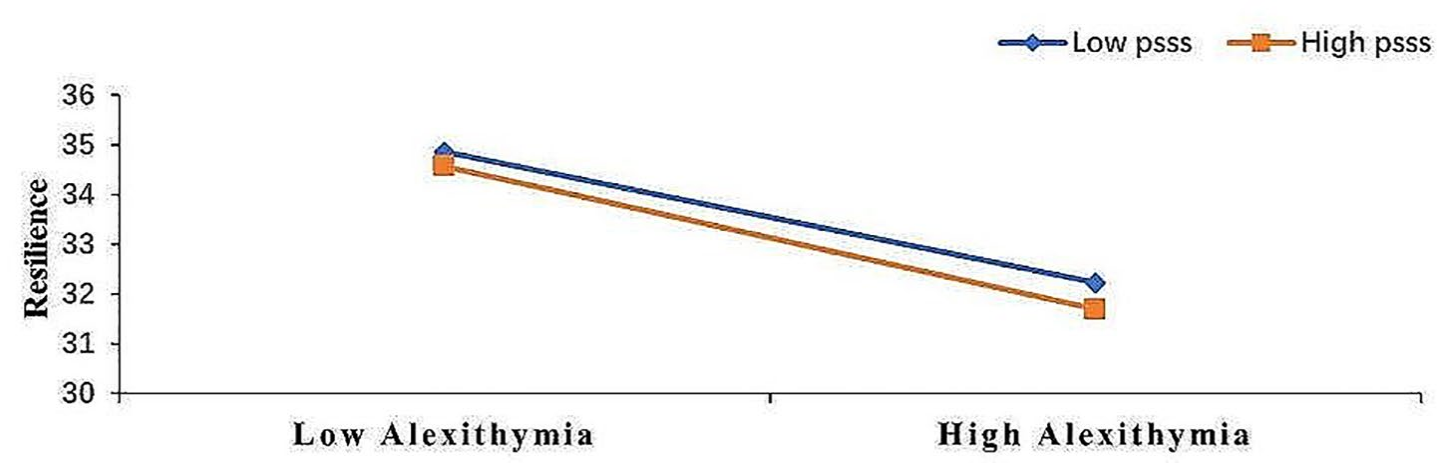
Perceived social support as a moderator of the relationship between alexithymia and psychological resilience

Further, simple slope analyses were conducted. Figure 2 shows that alexithymia was a psychological resilience (simple slope = -0.98, t = -5.91, *p* < 0.001) for subjects with low perceived social support (M–1SD). However, alexithymia predicted psychological resilience negatively to a lesser extent (simple slope = -0.62, t = -5.91, *p* < 0.001) for subjects with high perceived social support (M + 1SD) which suggests that alexithymia’s predictive effect on psychological resilience decreases with an increase in individuals’ perceived social support.

**Fig. 3:**
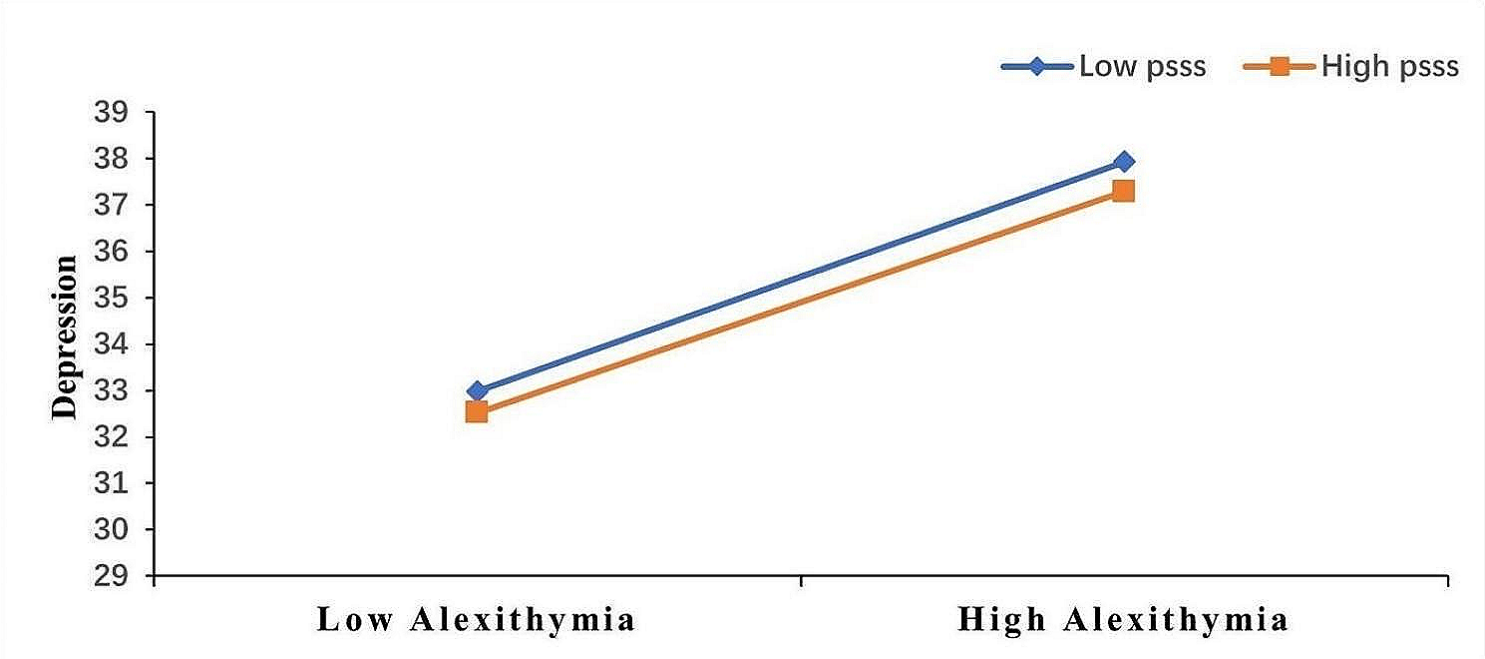
Perceived social support as a moderator of the relationship between alexithymia and depression

The simple slope analysis in Fig. 3 shows that alexithymia is a significant positive predictor of depression (simple slope = 0.97, t = 5.25, *p* < 0.001) for participants with lower perceived social support (M–1SD). However, it is a lower predictor of depression (simple slope = 0.48, t = 5.32, *p* < 0.001) for participants with higher perceived social support (M + 1SD). Thus, alexithymia’s predictive effect on depression reduced as individuals’ perceived social support increased.

Table 6 shows perceived social support as a moderator of the alexithymia–depression relationship. Psychological resilience’s mediating effect on depression was moderated by comprehension social support. This is evidenced by a significant increase in psychological resilience’s mediating role in moderating the narrative disorders–social anxiety relationship as the level of Perceived social support (moderating variable) increased.

**Table 6:** Understanding the direct and mediating effects of different social support levels

	PSSS	Effect	BootSE	BootLLCI	BootULCI
Direct effect	-12.42	0.42	0.03	0.35	0.48
	0.00	0.27	0.03	0.22	0.32
	12.42	0.12	0.03	0.06	0.19
Mediating effect of psychological resilience	-12.42	0.00	0.01	-0.01	0.01
	0.00	0.01	0.01	0.00	0.03
	12.42	0.03	0.01	0.00	0.06

*Note*. PSSS = Perceived Social Support Scale; SE = standard error; LLCI = lower limit confidence interval; ULCI = upper limit confidence interval

## Discussion

Recent research has found narrative traits in both patients with psychiatric disorders and healthy populations. However, relatively limited research has examined narrative disorders among university nursing students. Nursing students face various stressful events such as financial problems, academic pressure, and making new friends, and therefore, they are susceptible to psychological distress. This causes difficulties in recognising emotions, which leads to various negative emotional problems such as anxiety and depression, and affects nursing students’ physical and mental health. This is especially concerning considering the nursing shortage in the current workforce [65]. Therefore, nursing students should be assessed to identify dysphoria-related problems. Determining the mechanisms underlying narrative disorders and negative emotions is essential for developing preventive and interventional measures. This study builds on previous research by examining psychological resilience’s possible mediating role and comprehending perceived social support’s moderating role to further explore the interaction between narrative disorders and negative affect.

This study showed that alexithymia is significantly and positively correlated with anxiety, depression, and stress and negative affect, consistent with previous studies showing higher depression in patients with narcissism [66]. This may be due to the information age, nursing students’ increased use of social media, decreased frequency of face-to-face interactions, increased loneliness, and increased uncertainty about the future among young adults, all of which exacerbate nursing students’ expressive inhibition [67]. Other studies found that nearly 50% of patients with severe narrative dysphoria have difficulty recognising and expressing emotions (a rate significantly higher than 10% in the general population). This is likely because of poor emotion regulation and lack of self-control [68], difficulties in recognising and regulating emotional experiences, lack of expressive skills, lack of emotional regulation deficits, and inability to cope with stressful events. This leads to an accumulation of negative emotions in people with dysfunctional narratives [69].

Resilience is the ability to adapt to adversity, maintain equilibrium, stay in control, and deal with external stressors such as conflict [70]. Nursing students are at an important stage in their development as they have not yet reached professional status; lack autonomy, knowledge, and confidence; and face challenges associated with the healthcare environment [71]. Emotional intelligence and resilience are traits associated with skills needed to cope with these challenges, such as self-awareness, self-confidence, and stress management [72]. These can help students reconstruct negative or unfavourable experiences to build a sense of resilience. Resilience is correlated significantly negatively with dysphoria and negative emotions, and psychological resilience predicts negative emotions. Consistent with existing research, individuals with high-level resilience can cope better with negative emotions [73]. Other studies confirmed that individuals with dysphoria generally have low-level psychological resilience and cannot cope better with negative emotions [74]. This may be because low expressive skills in individuals with dysphoria have limited attention and memory processes, making it difficult to connect with external and sensory experiences and manage and report their feelings. In contrast, people with high resilience rarely feel helpless; they rely more on their own skills and can therefore better cope with negative emotions triggered by dysphoria. Some studies also recognised resilience as a state or ability [75]. It is feasible for people with high narrative dysphoria scores to gain new skills [76]. This highly negative correlation can be traced to differences in cognitive–emotion processing. Highly resilient individuals can better recognise stressful situations and assess their ability to effectively reduce stress realistically, which is positively associated with clinical communication [77]. Research indicates that students experience intense dissonance during externships, leading to profound negative emotions, if their psychological resilience is low [78].

A moderated mediation model was constructed to examine perceptual social support’s moderating role in the relationship between psychological resilience, affective disorders, and negative emotions. Perceptual social support moderated the alexithymia–negative affect relationship and the mediating chain of ‘alexithymia–psychological resilience–negative affect’. Individuals with relatively high perceived social support, especially from friends and partners, experience more care and warmth and receive more cognitive resources. Even if they cannot accurately identify others’ emotional needs, they can effectively mobilise cognitive resources to deal with stressful situations, improve self-efficacy, and reduce psychological stress, thus positively affecting their mental health [38]. Other studies showed that when individuals are under stress, perceived social support can relieve stress or help them adjust to emotional stress and reduce depressive symptoms [79].

Moreover, we found that alexithymia is more likely to decrease psychological resilience in individuals with low-level (vs. high-level) perceived social support, leading to depression during stressful events (including disasters and disease outbreaks). When individuals do not have adequate resilience and coping skills, they are more likely to suffer unfavourable mental and psychological consequences [80]. Support from peers, family, and friends helps individuals become adequately psychologically resilient to maintain emotional equilibrium when experiencing threatening and stress-inducing events. Thus, poor mental health outcomes, including depression, anxiety, and stress, are associated with social support. Adequate managerial and supervisory support as well as support from co-workers, peers, friends, and family are associated with reduced traumatic stress [81] and emotional distress [82]. Therefore, exploring perceived social support’s moderating effects on psychological resilience and depression can ameliorate the distress of negative emotions in individuals with dysphoria, particularly those with low perceived social support. This can effectively alleviate several psychological problems caused by dysphoria.

This study contributes to understanding alexithymia’s impact on nursing students’ negative emotions and changing relationships, with different levels of understanding of social support. However, it has several limitations. First, the cross-sectional study design could not validate the temporal order of the independent, mediating, moderating, and dependent variables, preventing causal inference. Because the findings have shown that negative emotions such as dysphoria and depression, anxiety, and stress can predict each other in longitudinal studies [83], we cannot rule out the possibility that negative emotions such as depression, anxiety, and stress may lead to higher levels of dysphoria through resilience as reverse causality. Future research should use longitudinal designs or experimental studies to explore the causal relationship between narrative dysfunction and negative affect through an aggregated cross-sectional design, multilevel linear modelling, or manipulation of independent and mediating variables. Additionally, future research using quasi-experimental or experimental designs is recommended to further assess the relationship between nurse practitioner dysphoria and depression, anxiety, and stress to enhance the understanding of the causality thereof. Second, this study was retrospective and participants may have recall bias. Third, the sample was not large enough to be representative, which affected the findings’ generalisability. As a result, a multicenter research is suggested for further study.Fourth, other psychological and personality variables such as neuroticism, which may influence negative affect, were not examined, which are also important background variables that may be associated with narrative disorders and depressive symptoms. Thus, further research needs to include more variables, and longitudinal designs and objective indicators could better substantiate the psychosocial mechanisms associated with narrative disorders that affect negative affect.

Despite these limitations, the findings provide insight into the mechanisms underlying negative emotions among nursing students and suggest possible prevention programmes. Attention should be paid to the stress students experience, and appropriate mental health counselling programmes should be developed in the curriculum to help reduce psychological distress. Educators and clinical administrators who treat depression should focus on cultivating nursing students’ ability to recognise and express emotions, improve psychological resilience, comprehend their level of social support, improve their ability to understand their own and others’ emotions, and better judge and cope with emotions to reduce patients’ negative emotional experiences. Thus, this moderated mediation model provides guidance for nursing students to improve their psychological resilience and social support and reduce negative emotional experiences by mitigating dysphoria. Such training will enhance their ability to recognise and manage their own and others’ emotions, improve psychological resilience to cope with stressful events by encouraging them to practice their ability to anticipate and manage frustrations related to personal goal setting or other work events, and increase navigational social support to moderate negative emotions. Further, positive thinking meditation can provide nursing students with negative emotions the opportunities to reduce stress and increase positive thinking [84].

## Conclusions

In summary, this is the first investigation of the relationship between alexithymia and depressive-anxiety stress in nursing students using a moderated mediation model. Resilience played a mediating role in the association between alexithymia and depressive-anxiety stress. Furthermore, the strength of mediation was moderated by perceived social support. The mediating role of psychological resilience in moderating narrative disorder-depression increased significantly with increasing levels of perceived social support (moderating variable). For nursing students, particularly those with low levels of perceived social support and resilience, it may be important to design interventions that combine increased resilience with increased perceived social support to reduce symptoms of depression anxiety stress.

### Relevance for clinical practice

The results provide useful information to policymakers and healthcare professionals, including psychiatric nurses, to design effective mental health improvement programmes for students. Mental health services in colleges and universities can be improved through counselling services; assistance; awareness programmes to inform students of the signs and symptoms of psychological problems and effective ways to cope with them; and implementation of campus intervention programs. Students with serious psychological problems should receive counselling to help them change their cognitive and behavioural patterns. Further studies should focus on recommending appropriate intervention models and strategies.

## Data Availability

Due to institutional review board protocols, the datasets generated and analysed for the current study are not publicly available but are available from the first author upon reasonable reques.

## Abbreviations

CD-RISC-10: 10-Item Connor-Davidson Resilience Scale
DASS: Depression Anxiety Stress Scale
PSSS: Perceived Social Support Scale
TAS-20: Toronto Alexithymia-20 Scale
