# Correction to: Alexithymia and negative emotions among nursing students: a moderated mediation model

**DOI:** 10.1186/s12912-025-04018-4

**Published:** 2025-10-21

**Authors:** Feifei Sun, Fang Wang, Xiaojing Hu, Jiaomei Xue, Shangkun Zheng, Jing Su, Qinghua Lu

**Affiliations:** 1 Department of Nursing, Shandong Mental Health Center, Shandong University, 49 Wenhua East Road, 250014 Jinan, Shandong China; 2 Xianning Vocational Technical College, 437100 Xianning, Hubei China; 3 Society and Law School, Shandong Women’s University, Changqing University Science and Technology Park, No. 2399, University Road, 25030 Jinan, Shandong China; 4 Human Resources Department, Shandong Mental Health Center, Shandong University, 49 Wenhua East Road, 250014 Jinan, Shandong China; 5 Editorial Board, Journal of Shandong First Medical University, No. 6699 Qingdao Road, Huaiyin District, 250000 Jinan, China; 6 Department of Infection Management, Shandong Mental Health Center, Shandong University, 49 Wenhua East Road, 250014 Jinan, Shandong China

**Correction to:** ***BMC Nurs*** ***23***, *167 (2024).* 10.1186/s12912-024-01832-0

Following the publication of the original article [1], it was noted that in the Ethical considerations section of this article, two instances of the term “patients” should have read “participants”.

The original article [1] has been corrected.
